# Increasing cyclic electron flow is related to Na^+^ sequestration into vacuoles for salt tolerance in soybean

**DOI:** 10.1093/jxb/erv392

**Published:** 2015-08-14

**Authors:** Yi He, Junliang Fu, Chenliang Yu, Xiaoman Wang, Qinsu Jiang, Jian Hong, Kaixing Lu, Gangping Xue, Chengqi Yan, Andrew James, Ligen Xu, Jianping Chen, Dean Jiang

**Affiliations:** ^1^State Key Laboratory of Plant Physiology and Biochemistry, College of Life Sciences, Zhejiang University, Hangzhou 310058, China; ^2^Department of Plant Sciences, University of Cambridge, Cambridge CB2 3EA, UK; ^3^Institute of Biotechnology, Zhejiang University, Hangzhou 310058, China; ^4^Laboratory of Plant Molecular Biology, Ningbo University, Ningbo 315211, China; ^5^State Key Laboratory Breeding Base for Zhejiang Sustainable Pest and Disease Control, Institute of Virology and Biotechnology, Zhejiang Academy of Agricultural Sciences, 310021 Hangzhou, China; ^6^CSIRO Agriculture Flagship, Queensland 4067, Australia

**Keywords:** ATP, CEF, Na^+^ accumulation, NDH complex, soybean.

## Abstract

In land plants, the NAD(P)H dehydrogenase (NDH) complex reduces plastoquinones and drives cyclic electron flow (CEF) around PSI. It also produces extra ATP for photosynthesis and improves plant fitness under conditions of abiotic environmental stress. To elucidate the role of CEF in salt tolerance of the photosynthetic apparatus, Na^+^ concentration, chlorophyll fluorescence, and expression of NDH B and H subunits, as well as of genes related to cellular and vacuolar Na^+^ transport, were monitored. The salt-tolerant *Glycine max* (soybean) variety S111-9 exhibited much higher CEF activity and ATP accumulation in light than did the salt-sensitive variety Melrose, but similar leaf Na^+^ concentrations under salt stress. In S111-9 plants, *ndhB* and *ndhH* were highly up-regulated under salt stress and their corresponding proteins were maintained at high levels or increased significantly. Under salt stress, S111-9 plants accumulated Na^+^ in the vacuole, but Melrose plants accumulated Na^+^ in the chloroplast. Compared with Melrose, S111-9 plants also showed higher expression of some genes associated with Na^+^ transport into the vacuole and/or cell, such as genes encoding components of the CBL10 (calcineurin B-like protein 10)–CIPK24 (CBL-interacting protein kinase 24)–NHX (Na^+^/H^+^ antiporter) and CBL4 (calcineurin B-like protein 4)–CIPK24–SOS1 (salt overly sensitive 1) complexes. Based on the findings, it is proposed that enhanced NDH-dependent CEF supplies extra ATP used to sequester Na^+^ in the vacuole. This reveals an important mechanism for salt tolerance in soybean and provides new insights into plant resistance to salt stress.

## Highlight

Enhanced cyclic electron flow contributes to increased accumulation of ATP in light, which facilitates Na^+^ sequestration into vacuoles of mesophyll cells mediated by up-regulation of genes associated with Na^+^ transport during salt stress.

## Introduction

Soil salinity represents an increasingly prominent problem in agriculture. It is estimated that at least one-third of the irrigated land in the world is now affected by salinity (Zhu, 2001; Munns and Tester, 2008). Because it is physically difficult to remove salt from the soil, improving crop tolerance to high salt becomes a critical task for breeders, and identifying molecular components conferring salt tolerance in plants can provide genetic markers for achieving this goal. Salinity affects plant growth by inducing secondary stresses such as metabolic toxicity, attenuated nutrient acquisition, membrane disorganization, accumulation of reactive oxygen species (ROS), and inhibition of photosynthesis (Hasegawa *et al.*, 2000). Meanwhile, salt injury to plants is caused by ionic toxicity that is specific to a particular ion (such as Na^+^) and by osmotic stress (Yeo, 1998; Hasegawa *et al.*, 2000).

Salt tolerance in plants requires three interconnected cellular functions that (i) prevent or alleviate damage, (ii) re-establish homeostatic conditions in the new, stressful environment, and (iii) resume growth, albeit at a reduced rate (Zhu, 2001). Although salt-tolerant plants display complex molecular responses, these processes generally consume ATP. In plants, ATP is one of the most important products of photophosphorylation, which is a highly regulated process that integrates different electron transfer pathways to convert light energy into ATP and NADPH and balance this production of chemical energy with its utilization (Johnson *et al.*, 2014). In addition, photosynthesis is the most important source of plant dry matter production. Accordingly, maintaining a high photosynthetic rate is important to protect the photosynthetic apparatus from damage by excess light energy and ROS under stress conditions.

During plant evolution, the photosynthetic thylakoid membrane systems developed mechanisms to adapt to stress conditions (Munné-Bosch *et al.*, 2005; Takabayashi *et al.*, 2009). One of the available pathways for photoprotection is to accelerate cyclic electron flow (CEF) around PSI (Shikanai *et al.*, 1998; Horváth *et al.*, 2000; Munekage *et al.*, 2004). Plants have two known pathways for CEF: the protein gradient regulation 5 (PGR5)/PGR-like 1 (PGRL1)–ferredoxin (Fd)-dependent pathway and the NAD(P)H dehydrogenase (NDH) complex-dependent pathway (Johnson *et al.*, 2014). Examination of mutants in *Arabidopsis thaliana* showed that efficient photosynthesis requires PGR5/PGRL1–Fd-dependent CEF (Munekage *et al.*, 2004). In contrast, knockout *crr* mutations (impaired NDH activity) do not affect overall photosynthetic electron transport and phenotypes (Munekage *et al.*, 2004), indicating that NDH complex-mediated CEF may not participate in normal photosynthetic processes. Instead, many studies have demonstrated that NDH complex-mediated CEF may play a critical role in protecting the photosynthetic apparatus under stress conditions (Wang *et al.*, 2006; Yang *et al.*, 2007; Huang *et al.*, 2011). The amounts of the NDH complex significantly increase in *Hordeum vulgare* (barley) under photo-oxidative and osmotic stress (Casano *et al.*, 2001; Guéra *et al.*, 2005) and in *Avena sativa* (oat) under high light stress (Quiles, 2006). In addition, increased amounts of NDH complex in high mountain plant species may enhance adaptation to high light intensity and low temperature (Streb *et al.*, 2005). Moreover, the NDH complex has been suggested to supply extra ΔpH of trans-thylakoid membranes and additional ATP for optimal photosynthesis under stress conditions when CO_2_ assimilation is limited (Wang *et al.*, 2006; Shikanai, 2007). These results support the hypothesis that the NDH complex contributes to enhancing photosynthesis and stress tolerance under stress conditions. However, how the NDH complex might function in alleviating salt stress injury to plants, and its relationship to ATP biosynthesis from NDH-dependent CEF and to Na^+^ accumulation in the leaf all remain controversial.

It was previously reported that S111-9 soybean, a somatic hybrid descended from wild soybean ACC547 (cytoplasm donor) and Melrose cultivar (nucleus donor), partially inherited salt stress tolerance, maintaining a higher net photosynthetic rate and increased CEF in leaves compared with controls at the same leaf Na^+^ concentration (Yang *et al.*, 2007). It was also found that Na^+^ accumulated in mesophyll cells of S111-9 and Melrose. High concentrations of Na^+^ in plant cells inhibit the activities of many enzymes (Tester and Davenport, 2003), so it is important to prevent Na^+^ from accumulating in cytoplasm and organelles other than the vacuole. These observations support the possibility of a mechanism that increases Na^+^ accumulation in the vacuole of salt-tolerant soybean S111-9.

The aim of this study was to clarify whether NDH-dependent CEF contributes to ATP biosynthesis, Na^+^ transport, and Na^+^ accumulation in mesophyll cells. The findings indicated that in S111-9 under salt stress, but not in Melrose, enhanced NDH-dependent CEF produces additional ATP, which is required for Na^+^ accumulation in the vacuole.

## Materials and methods

### Plant growth conditions

The salt-sensitive cultivar Melrose (*Glycine max*) and the salt-tolerant line S111-9, a stable line selected from somatic hybrid descendants of wild salt-tolerant ACC547 (*G. cyrtoloba*; Yang *et al.*, 2007), were used in this study. When the first pair of leaves was fully expanded, the seedlings were transplanted into complete nutrient solution and cultured at 25/22 °C (12h light/12h dark) with ~70% relative humidity in a growth chamber. For experiments, 25-day-old plants were selected and transferred to complete culture solution (He *et al.*, 2014; Wang *et al.*, 2015) with 0 or 150mM NaCl. The third fully expanded leaves were used for all analyses.

### Visualization of Na^+^ ions

The third leaves of 25-day-old plants after salt treatment were cut into pieces (~5×2mm), which were fixed on the plates with 2.5% (w/v) agar. Then, agar blocks with leaf pieces were cut from plates and placed on a vibratome for preparation of 140 μm thin sections. The sections were stained with 20 μM CoroNa Green AM (Invitrogen) in the presence of a final concentration of 0.02% (w/v) pluronic acid (Invitrogen) for 3h (Oh *et al.*, 2009). Where indicated, 5 μM FM4-64 (Invitrogen) was added after incubation with CoroNa Green (incubation for ~20–30min with FM4-64). The palisade layer cells were selected for observation and all sections were observed by confocal microscopy (Zeiss LSM-710).

### Elemental analysis

Tissue samples were collected and dried at 65 °C for 72h to yield ~0.5g of tissue for elemental analysis. After cooling, each sample was weighed and 0.1g was used for analysis. All samples were digested with 6ml of concentrated HNO_3_ (68%, Sangon). After digestion, all samples were diluted to 50ml with 18 MΩ water. The leaf, stem, and root tissue concentrations of Na^+^, K^+^, and Ca^2+^ were measured by inductively coupled plasma mass spectrometry (ICP-MS) as described previously (Baxter *et al.*, 2010). All samples were normalized to calculated weights, as determined with the solution concentrations.

### Measurement of chlorophyll fluorescence and redox changes of P700

The plants were adapted in the dark for 30min prior to measurement. Chlorophyll (Chl) fluorescence and redox changes of P700 were performed using a Dual-PAM 100 Chl fluorescence analysr (Heinz Walz, Effeltrich, Germany) as described in Wang *et al.* (2006). The post-illumination transient increase in Chl fluorescence was determined according to the procedure described previously (Mi *et al.*, 1995; Shikanai *et al.*, 1998). Soybean plants were kept at 25 °C and third intact leaves were used for measurement, which were also utilized for assessment of redox changes of P700. Weak modulated irradiation (<0.1 μmol m^–2^ s^–1^) was used to determine *F*_o_. Subsequently, a saturating light (>7000 μmol m^–2^ s^–1^) flash was given to determine *F*_m_, and then actinic light (1000 μmol m^–2^ s^–1^) was turned on. After 3min, the actinic light was turned off and fluorescence yield changes were continuously recorded (Yang *et al.*, 2007). Redox changes of P700 were monitored by absorbance changes at 810–830nm, and the *t*_1/2_ of P700^+^ re-reduction following far-red light (>705nm, 5.2 μmol m^–2^ s^–1^) exposure for 1min was calculated (Klughammer and Schreiber, 1998).

### Determination of ATP content

To determine ATP content in leaves, the third leaves were collected and 0.1g samples were placed in 10ml tubes, with 2ml of Tris–HCl (pH 7.8). The tubes with samples were kept for 10min at 100 °C in a boiling water bath for ATP extraction. Then, the samples were kept at room temperature for cooling and 10 μl of ATP extraction solution was used for analysis. The ATP standard curve was obtained using standard ATP samples in an ENLITEN ATP Assay Kit (Cat. FF2000, Promega) by a GloMax 20/20 Luminometer (Promega). The ATP content of all samples was calculated via the ATP standard curve, as determined with the weight of samples and solution concentrations. The detailed procedures were performed following the instructions in the ENLITEN ATP Assay System.

### Antibody preparation

The full-length *ndhH* sequence was cloned by PCR from cDNA with the primers 5′-*GGATCC*ATGAATATCTCAACTACAAGA-3′ and 5′-*GTCGAC*TCAACGATCAACTTCTCCCA-3′ (underlining indicates *Bam*HI and *Sal*I sites, respectively). The amplified fragment was ligated into pGEX-4T-2 vector for production of glutathione *S*-transferase and the thrombin target at the N-terminus of NDH-H. *Escherichia coli* BL21 (DE3) transformed with the plasmid was incubated at 37 °C in LB medium. Expression of the recombinant protein was induced by addition of 1mM isopropyl-β-d-thiogalactopyranoside at OD_650_ between 0.4 and 0.6, and cells were grown for 3h. Cells were bulked and resuspended in buffer A [50mM Tris–HCl, pH 7.5, 0.3M NaCl, 7mM β-mercaptoethanol, and 1mM PMSF (phenylmethylsulphonyl fluoride)]. The subsequent steps were performed at 4 °C. Sonication was used to disrupt the cells, and the samples were centrifuged at 15 000 *g* for 30min to remove cell debris. The supernatant was loaded onto a 1ml GSTrap HP column (GE Healthcare Biosciences) that had been equilibrated with phosphate-buffered saline (PBS) buffer (0.01mol l^–1^ PBS pH 7.4) containing 10mM imidazole. The column was washed with PBS buffer containing 30mM imidazole. The recombinant protein was eluted with 0.2M glycine-HCl (pH 3.0), analysed for purity by SDS–PAGE, and injected into rabbits for antibody production. For anti-NdhB, a synthesized peptide of 15 amino acids (DLTSDQKDISWFYFC) was directly injected into rabbits for antibody production. Anti-NDH-B and anti-NDH-H were confirmed as specifically binding antibodies by immunoblotting.

### Fixation and immunolocalization

The subcellular location of NDH-B and NDH-H was determined by immunogold labelling as described by He *et al.* (2014), using the antibodies specific for NDH-B or NDH-H (described above). Micrographs at ×40 000 magnification with clear chloroplast structure and no large starch granules were selected for analysis. A two-dimensional grid of 0.04 μm^2^ divided each picture (~345 grid units) and the grid units entirely on chloroplast were selected to count gold particles. The labelling density was determined by counting gold particles and calculating the number per unit area (μm^2^). Labelling density was analysed in 7–8 individual cells of palisade layers from different immune-labelled sections of each variety.

### Immunoblotting and RNA gel blotting

NdhB and NdhH proteins were analysed by immunoblotting, following a procedure modified from a previously described method (He *et al.*, 2014). Purified protein samples (30 μg each) were separated by 12% (w/v) SDS–PAGE and transferred to polyvinylidene fluoride (PVDF) membranes. The PVDF membranes were incubated with a primary antibody against NDH-B (1:2000 dilution) or NDH-H (1:2500 dilution). The final detection was performed using secondary fluorescent anti-rabbit IgG, following the instructions of the SuperSignal West Pico Chemiluminescent Western blotting Kit (Thermo Scientific).

Total RNA was extracted from S111-9 or Melrose using the RNAsio Reagent (TaKaRa). RNA gel blotting hybridization for mRNA expression analysis was performed using the method described by Du *et al.* (2007). Hybridization probes were prepared by end labelling with biotin (Invitrogen), and the sequences of the hybridization probes (*ndhB*: 5′-GGAATTGTTTGAT CTTAAAGGGGGCCTT CGATAATTTCGC-3′; *ndhH*: 5′- GGGTTTGTGCAC CAATATCT GCCATAAA AGGTCCTAGCC-3′) are completely complementary to *ndhB* and *ndhH*, respectively.

### Quantitative real-time PCR

RNA was extracted from three biological replicates of 25-day-old soybean plants using RNAsio Reagent (TaKaRa). cDNA was synthesized from 1 μg of DNase-treated RNA with the TaKaRa First Strand cDNA Synthesis Kit using oligo(dT) primers. Technical replicates of gene-specific products were amplified in 10 μl reactions using the Rotor-Gene SYBR Green PCR Kit on a Rotor-Gene 6000 Real-Time PCR machine fitted with a Rotor-Disc 72 (QIAGEN). Primer sequences are described in Supplementary Table S2 available at *JXB* online. Relative transcript levels were determined by incorporating PCR efficiencies as described previously (Talke *et al.*, 2006).

### Measurements of vacuolar H^+^-ATPase activity

The V-ATPase activity was determined by the detailed method described by Tang *et al.* (2012). Leaf microsomal fractions were prepared from 25-day-old hydroponically grown plants treated or not with thenoyltrifluoroacetone (TTFA). Plant materials were ground with cold homogenization buffer containing 350mM sucrose, 70mM Tris–HCl (pH 8.0), 3mM Na_2_EDTA, 0.2% (w/v) BSA, 1.5% (w/ v) polyvinylpyrrolidone (PVP)-40, 5mM DTT, 10% (v/v) glycerol, 1mM PMSF, and 1× protease inhibitor mixture (Roche). The homogenate was filtered through four layers of Microcloth (Calbiochem, Cat. 475855) and centrifuged at 4000 *g* for 20min at 4 °C. The supernatant was filtered through Microcloth again and then centrifuged at 100 000 *g* for 1h. The resulting microsomal pellet was resuspended in 350mM sucrose, 10mM Tris–MES (pH 7.0), 2mM DTT, and 1×protease inhibitor mixture. The V-ATPase activity of 10 μg of microsomal membranes was determined as phosphate (Pi) release after 40min incubation at 28 °C. V-ATPase activity was calculated as the value difference between the measurements in the absence and the presence of 200nM concanamycin A plus 50mM NaNO_3_. The reaction was colorimetrically examined at 355nm.

### Statistical methods

All assays described above were repeated at least three times on three biological replicates. For multiple comparisons, the data first were examined by one-way ANOVA to check the equality of variance (Levene test), and then Tukey’s multiple comparison tests were used to determine the significant difference (*P*<0.05) of means with the SAS 8.0 statistical software package (SAS Institute, Inc., Cary, NC, 2000; He *et al.*, 2014).

## Results

### Effect of salt stress on phenotype and the accumulation of Na^+^ in mesophyll cells

**Fig. 1.**
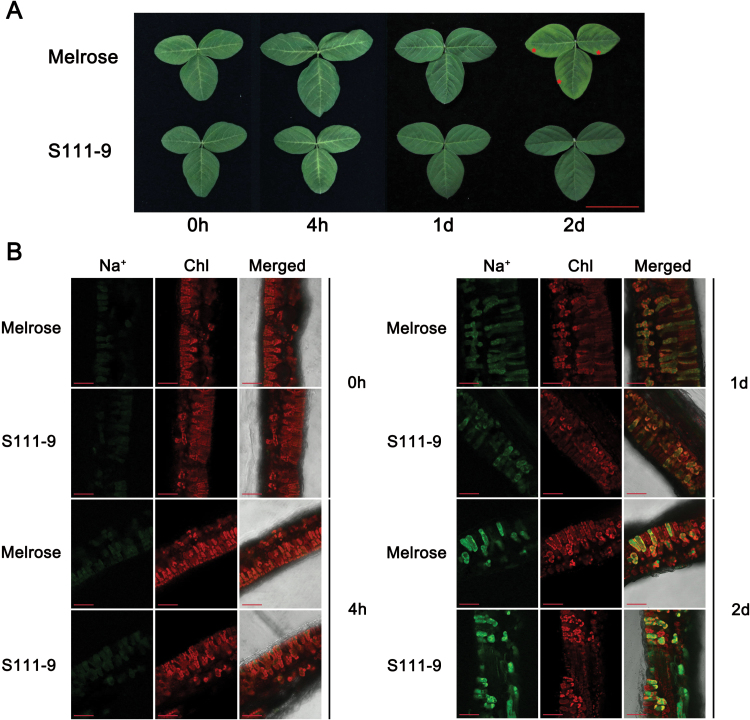
Effects of salt stress on phenotype and the distribution of Na^+^ in soybean leaves. (A) Phenotypes of the third leaves in Melrose and S111-9 under 150mM NaCl stress for 0h, 4h, 1 d, and 2 d. Scale bar=5cm. (B) Cellular localization and distribution of Na^+^ in Melrose and S111-9 under 150mM NaCl stress for 0h, 4h, 1 d, and 2 d. The third leaves of 25-day-old plants were sectioned and stained with CoroNa Green AM to report Na^+^ concentration. All sections were observed at ×20 magnification. Scale bars=50 μm.

To compare the salt resistance of different genotypes, S111-9 and Melrose soybean plants were cultured in nutrient solution with 150mM NaCl. After 2 d of salt stress, chlorosis and necrotic lesions appeared in the leaves of Melrose but not of S111-9 (Fig. 1A; see red asterisks). Melrose plants also lost more water than did S111-9 (Supplementary Fig. S1 at *JXB* online) and showed visible wilting after salt stress. Staining with CoroNa Green, an indicator of Na^+^ concentration, showed that although the fluorescence signal intensity increased significantly in both varieties upon salt stress (Fig. 1B), there was no obvious different between both varieties. Together, the above results provided further evidence that S111-9 had higher tolerance to salt stress than Melrose.

### Changes in the concentration and distribution of Na^+^, K^+^, and Ca^2+^ in the whole plant

**Fig. 2.**
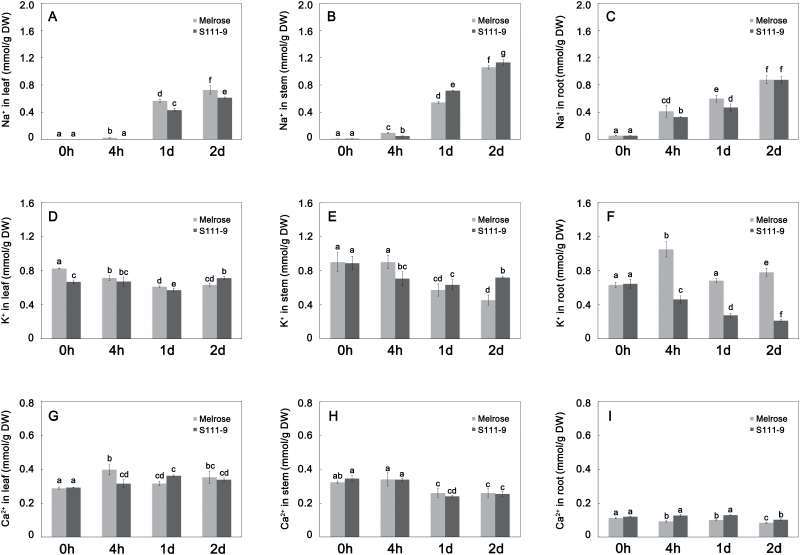
Effects of salt stress on Na^+^, K^+^, and Ca^2+^ in leaf, stem, and root. Concentrations of Na^+^ (A–C), K^+^ (D–F), and Ca^2+^ (G-I) in leaf (A, D, G), stem (B, E, H), and root (C, F, I). The concentrations of Na^+^, K^+^, and Ca^2+^ were measured in dry samples by ICP-MS. All data were normalized to calculated weights, as determined with the solution concentrations. Bars represent the mean ±SD of at least three independent experiments. Different letters indicate significantly different values (*P*<0.05) by Tukey’s test.

The concentrations of Na^+^, K^+^, and Ca^2+^ were measured by ICP-MS. After 4h salt stress, the Na^+^ concentration increased in roots from ~0.05 mmol g^–1^ DW to 0.4 mmol g^–1^ DW in both varieties (Fig. 2C). Although the S111-9 roots had a significantly lower Na^+^ concentration than the Melrose roots at 4h and at 1 d salt stress, the two varieties showed no difference after 2 d salt stress. The concentrations of Na^+^ in leaves and stems also increased significantly in both varieties after 1 d salt stress. Although the Na^+^ concentration of Melrose leaves was significantly higher than that in S111-9, the difference in absolute values became smaller after 2 d treatment. However, stems showed the opposite tendency, with S111-9 showing a larger increase than Melrose (Fig. 2A, B), to 1.13 mmol g^–1^ DW and 1.06 mmol g^–1^ DW in the stems of S111-9 and Melrose, respectively (Fig. 2B) after 2 d stress. After 2 d salt stress, the leaf K^+^ concentration in Melrose decreased significantly compared with that in S111-9, and the leaf K^+^ concentration in S111-9 returned to the level observed at 4h salt stress (Fig. 2D). This change in K^+^ concentration affected the K^+^/Na^+^ ratio (Supplementary Fig. S2 at *JXB* online) and might thus alter Na^+^ transport and accumulation in vacuoles of mesophyll cells. The K^+^ concentration in stems and leaves of both varieties showed the same trends, but, after salt treatment, Melrose roots had a much higher K^+^ concentration than did S111-9 roots. The Ca^2+^ concentration in leaves (Fig. 2G) also significantly increased in both varieties after salt treatment, but it decreased in stems and roots (Fig. 2H, I) after 1 d or 2 d salt stress. Regression analysis showed a significant negative correlation between Na^+^ and K^+^ in leaf and stem of Melrose, in roots of S111-9, and between Na^+^ and Ca^2+^ in stems and roots of Melrose and in stems of S111-9 (Supplementary Table S1).

### Effect of salt stress on NDH activity and ATP contents

**Fig. 3.**
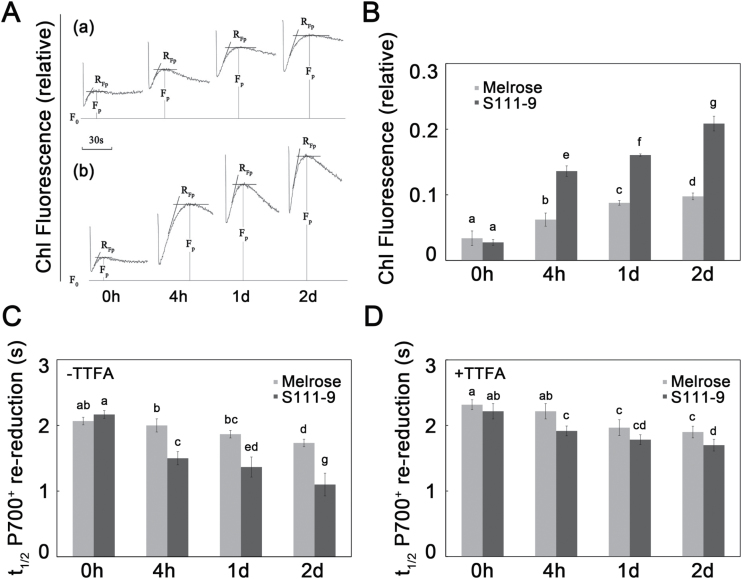
Effects of salt stress on NDH-dependent CEF activity. The third fully expanded leaves of Melrose and S111-9 under 150mM salt stress for 0h, 4h, 1 d, and 2 d were used in this experiment after 30min dark adaptation. (A) A typical post-illumination transient increase induction curve in Melrose (a) and S111-9 (b) under 150mM salt stress. *F*_0_, dark fluorescence level; *F*_p_, the height of the post-irradiation fluorescence increase. (B) The effects of salt stress on the post-illumination fluorescence increase in Melrose and S111-9. (C) The effects of salt stress on the *t*_1/2_ of P700^+^ re-reduction. The typical trace of the dark re-reduction of P700^+^ was measured following illumination by far red light (>705nm, 6 μmol m^–2^ s^–1^) in the absence of 100 μM TTFA (an inhibitor that binds ferredoxin). (D) The effects of salt stress on the *t*_1/2_ of P700^+^ re-reduction in the presence of 100 μM TTFA. Bars represent the mean ±SD of six independent experiments. Different letters indicate significantly different values (*P*<0.05) by Tukey’s test.

NDH activity was measured *in vivo* by measuring the post-illumination transient increase in Chl fluorescence after turning off actinic light, an increase that is due to NDH-dependent reduction of the plastoquinone pool in darkness (Munekage *et al.*, 2004). The results showed no difference in the increase in Chl fluorescence between the two varieties before salt stress (Fig. 3A, B). However, the increase rapidly became greater in both S111-9 and Melrose after salt stress. Compared with the unstressed plants, the increase of Chl fluorescence was ~6-fold more in S111-9, but only 2-fold more in Melrose after 2 d salt stress. These results indicated that under salt stress, NDH-dependent CEF activity increased faster in salt-resistant S111-9 plants than in salt-sensitive Melrose plants.

The dark re-reduction of P700^+^ is often used as another indicator of CEF (Burrows *et al.*, 1998; Nakamura *et al.*, 2013). The dark re-reduction of P700^+^ was measured and the *t*_1/2_ of P700^+^ re-reduction was calculated in both varieties after treatment with 150mM NaCl in the absence and the presence of 100 μM TTFA (an inhibitor of NDH-mediated plastoquinone reduction in cyanobacteria and *Chlamydomonas reinhardtii*) (Godde, 1982; Mi *et al.*, 1994) (Fig. 3C, D). Under salt stress, there was a greater decrease in the *t*_1/2_ of P700^+^ re-reduction in S111-9 (going from 2.17 s without salt stress to 1.10 s with salt stress) than in Melrose (going from 2.07 s to 1.73 s) in the absence of 100 μM TTFA (Fig. 3C), and there was a significant difference between the varieties. Although there was still a difference between the varieties in the presence of 100 μM TTFA, the difference (going from 2.22 s to 1.70 s in S111-9 and from 2.32 s to 1.90 s in Melrose) was obviously smaller than that in the absence of 100 μM TTFA, which implied that the CEF was attenuated by TTFA. This provided more evidence that salt stress stimulated CEF much more in S111-9 than in Melrose, and TTFA could partly inhibit the CEF activity.

**Fig. 4.**
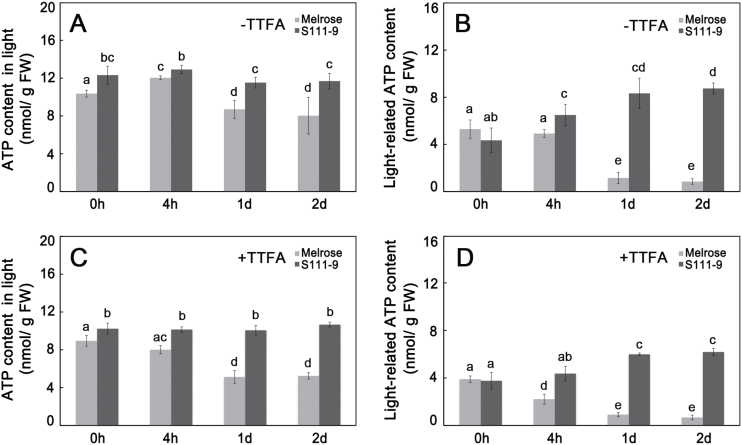
ATP contents of leaves under 150mM salt stress. The leaves used for these measurements were the same as those used for the Chl fluorescence and dark re-reduction of P700^+^ analysis shown in Fig. 3. All data were determined in Melrose and S111-9 under salt stress after 0h, 4h, 1 d, and 2 d. (A) ATP content in light in the absence of 100 μM TTFA (an nhibitor that binds ferredoxin); (B) light-related ATP content in the absence of 100 μM TTFA, as calculated by subtracting the ATP content in darkness from the ATP content in light. The samples for ATP content in darkness were collected and kept in darkness for 24h, and reflect the ATP produced by oxidative phosphorylation. The light-related ATP content reflects the ATP accumulation produced by photophosphorylation. (C) ATP content in light in the presence of 100 μM TTFA; (D) light-related ATP content in the presence of 100 μM TTFA. Bars represent the mean ±SD of four independent experiments. Different letters indicate significantly different values (*P*<0.05) by Tukey’s test.

To investigate whether enhanced CEF could promote ATP production, ATP contents were measured in the light, which reflects ATP produced by both photosynthetic and oxidative phosphorylation, and in darkness, which mainly reports ATP produced by oxidative phosphorylation. Therefore, the difference between the light and dark ATP production represents the ATP accumulation from photophosphorylation. In light conditions, S111-9 showed significantly higher ATP contents than Melrose after salt stress (Fig. 4A). ATP production remained stable in S111-9 (~12 nmol g^–1^ FW), and decreased significantly in Melrose after 1 d and 2 d salt stress (Fig. 4A). The difference in ATP accumulation in light compared with in darkness was much greater in S111-9 than in Melrose (Fig. 4B). Moreover, the light-mediated portion of the ATP content of S111-9 rose markedly to 8.7 nmol g^–1^ FW after 2 d salt stress, whereas it significantly decreased in Melrose (Fig. 4B) after salt stress. Additionally, in the presence of 100 μM TTFA, ATP content in the light and light-related ATP content were decreased by ~15–25% (Fig. 4C, D). All of these findings indicated that the increased ATP content in the light might be produced by cyclic photophosphorylation via enhanced CEF in S111-9.

### Effect of salt stress on the protein and mRNA abundance of NDH-B and NDH-H

**Fig. 5.**
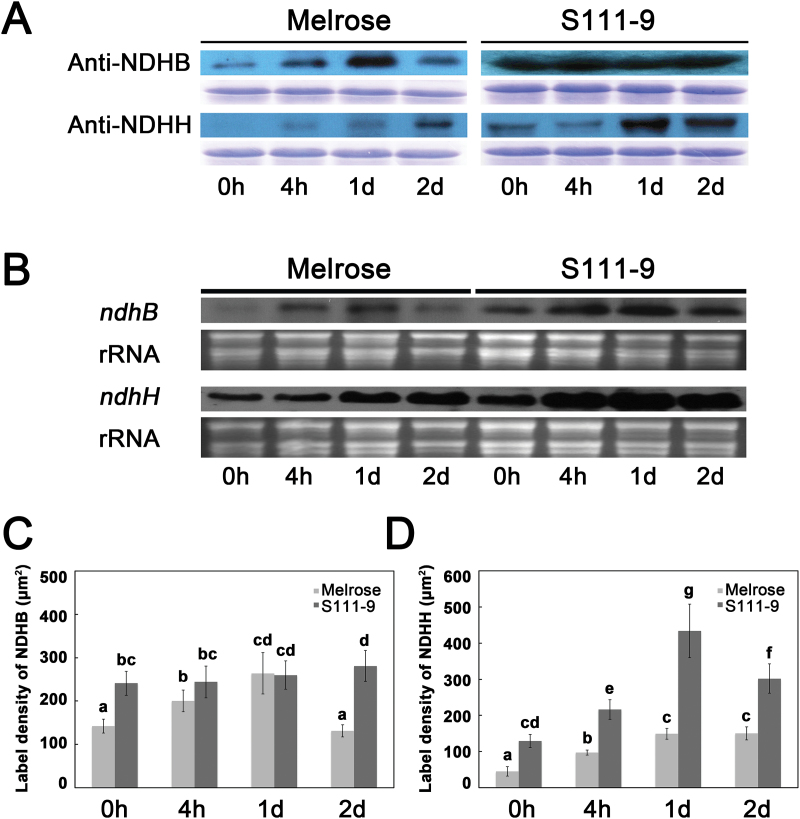
Effect of salt stress on the protein and mRNA abundance of *ndhB* and *ndhH*. (A) Changes in NDH-H and NDH-B content were analysed using immunoblotting in the leaves of Melrose and S111-9 treated with 150mM NaCl for 0h, 4h, 1 d, and 2 d. Equal loading is shown by Coomassie-stained gels below each blot. (B) Effect of salt stress on the mRNA expression of *ndh*B and *ndh*H. RNA gel blotting hybridization analysis of *ndh*B and *ndh*H was performed using the total RNA from Melrose and S111-9 treated with 150mM NaCl stress for 0h, 4h, 1d, and 2 d. The RNA samples loaded were visualized by ethidium bromide staining of 28S rRNA (lower panel). (C and D) Immunogold labelling of NDH-B and NDH-H in the chloroplast organelles of the mature leaves of soybean (*Glycine max*) under 150mM salt stress. Seven to eight individual cells of palisade and spongy layers were examined in several immunolabelled sections. Numbers of gold particles per unit area (μm^2^) are given as mean ±SD. (C) Effect of salt stress on label density of NDH-B (μm^2^). (D) Effect of salt stress on label density of NDH-H (μm^2^). Different letters indicate significantly different values (*P*<0.05) by Tukey’s test.

It was further investigated whether the increased ATP content in S111-9 under salt stress could be attributed to enhanced CEF caused by increased accumulation of the NDH complex. The amounts of NDH-B and NDH-H are often used to monitor the accumulation of the NDH complex (Hashimoto *et al.*, 2003; Munshi *et al.*, 2005). Accordingly immunoblotting was performed with antibodies raised against NDH-B and NDH-H to estimate the amount of NDH complex in S111-9 and Melrose. As shown in Fig. 5A, the constitutive levels of NDH-B and NDH-H proteins were much higher in S111-9 than in Melrose. Both NDH-B and NDH-H protein levels were induced by salt stress in Melrose. In S111-9, a salt-mediated increase was observed only in NDH-H (Fig. 5A). During the 2 d salt treatment, NDH-B and NDH-H protein levels were generally higher in S111-9 than in Melrose. Moreover, the results of immunogold labelling for NDH-B and NDH-H protein (Fig. 5C, D) are in accordance with the above immunoblotting analyses. Furthermore, the relative mRNA abundance of *ndhB* and *ndhH* was higher in S111-9 than in Melrose, both before and after salt stress (Fig. 5B), especially for *ndhH*, consistent with the results of immunoblotting. The mRNA levels of *ndhB* and *ndhH* were up-regulated by salt stress in both Melrose and S111-9. These findings implied that in S111-9, the higher abundance of the NDH complex might result in faster CEF and more ATP accumulation in the light.

### Differences in the distribution of Na^+^

**Fig. 6.**
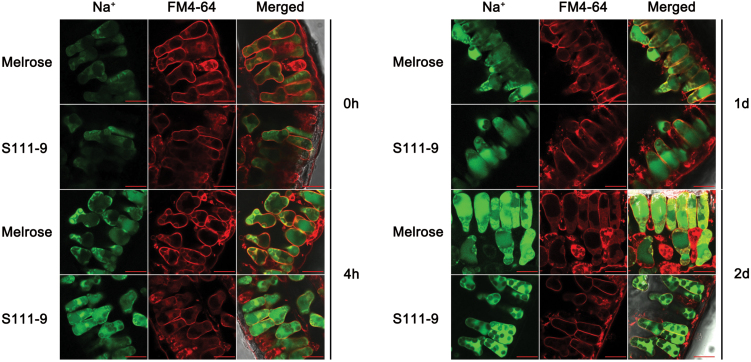
Distribution and accumulation of Na^+^ in soybean leaves. Comparison of the distribution and accumulation of Na^+^ in the leaves of Melrose and S111-9 treated with 150mM salt for 0h, 4h, 1 d, and 2 d. Confocal images of leaf sections stained with CoroNa Green (green) and FM4-64 (red) are shown. CoroNa Green fluorescence intensity indicates the Na^+^ content. FM4-64 is an endocytotic marker dye that is internalized to the tonoplast via the endosomal compartment, and red fluorescence indicates the location of the vacuole. All sections were observed at ×63 magnification. Scale bars=20 μm.

**Fig. 7.**
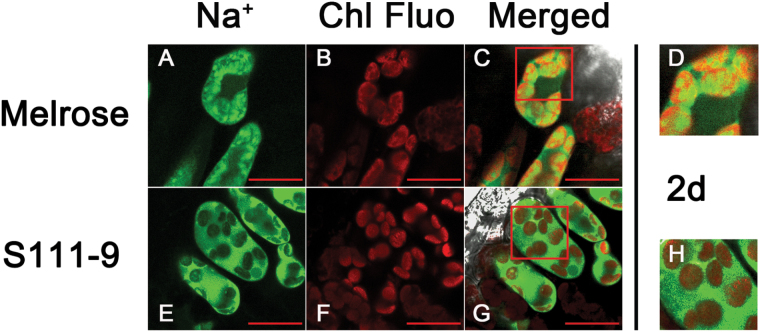
Localization of Na^+^ in the leaves of Melrose and S111-9. Comparison of the localization of Na^+^ to chloroplasts of Melrose (A–D) and S-1119 (E–H) after 150mM salt stress for 2 d. Confocal planes of leaf sections with Chl autofluorescence (red) and stained with CoroNa Green (green) are shown. CoroNa Green fluorescence and Chl fluorescence intensities indicate the Na^+^ content and the location of chloroplasts, respectively. (A and E) CoroNa Green fluorescence; (B and F) Chl fluorescence; (C and G) merged images; (D and H) close-ups of the boxed regions of (C) and (G), respectively. All sections were observed at ×63 magnification. Scale bars=20 μm.

To understand whether differences in the distribution of Na^+^ in mesophyll cell organelles affect salt tolerance in the two varieties, CoroNa Green was used to reveal the distribution of Na^**+**^ in mesophyll cells. FM4-64 was used to show vacuolar membranes, as it specifically stains vacuolar membranes (Kutsuna and Hasezawa, 2002). It was found that Na^**+**^, as measured by CoroNa fluorescence intensity, increased remarkably with salt stress, and enhanced Na^**+**^ was mainly concentrated in vacuoles after 1 d and 2 d salt stress (Fig. 6; Supplementary Fig. S3 at *JXB* online). After 2 d salt stress, the Melrose mesophyll cells showed little recognizable vacuolar membrane staining, indicating that the membrane was damaged. These results, combined with Na^**+**^ and Chl fluorescence data, further indicated that most of the Na^+^ was concentrated in vacuoles. However, co-fluorescence of Na^+^ and chloroplasts was observed in mesophyll cells of Melrose leaves (Fig. 7D), in comparison with S111-9, which showed little or no co-fluorescence (Fig. 7H). These observations indicate that the entry of Na^+^ into chloroplasts was more common in Melrose than in S111-9.

### Effect of salt stress on vacuolar H^+^-ATPase activity

**Fig. 8.**
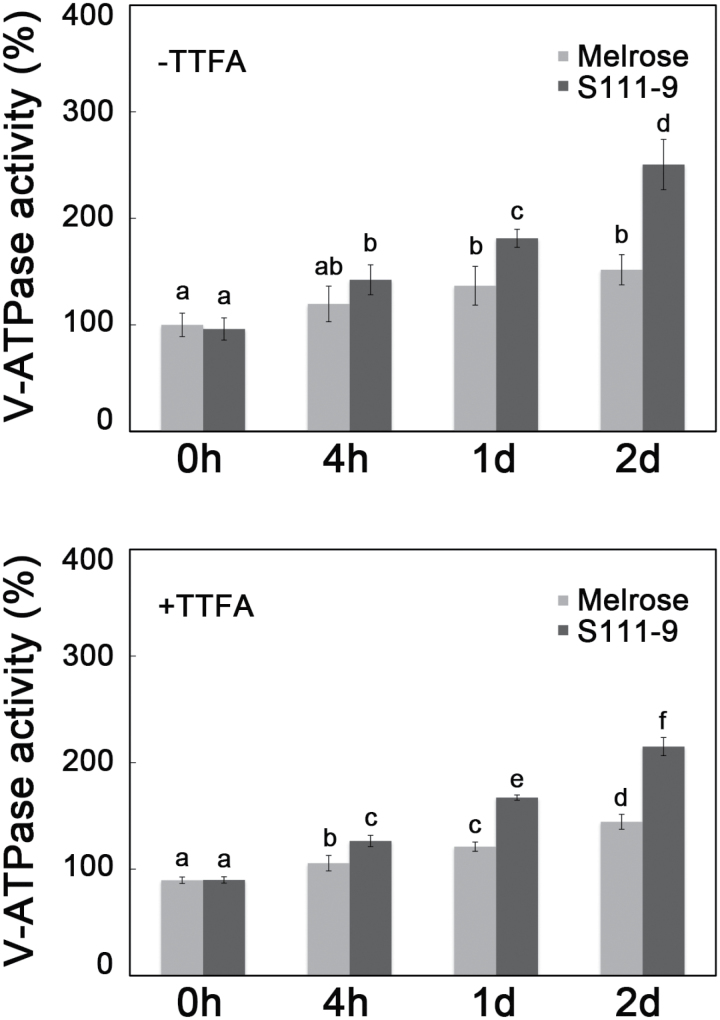
Effects of salt stress on vacuolar H^+^-ATPase activity. Vacuolar H^+^-ATPase hydrolytic activity was determined from microsomal membranes of 25-day-old plants of Melrose and S111-9 treated with 150mM NaCl for 0h, 4h, 1 d, and 2 d in the absence and the presence of 100 μM TTFA. Results are shown as a percentage of the Melrose 0h activity without TTFA. Values are the mean ±SD at least of three replicate experiments. (A) Vacuolar H^+^-ATPase hydrolytic activity in the absence of 100 μM TTFA; (B) vacuolar H^+^-ATPase hydrolytic activity in the presence of 100 μM TTFA. Different letters indicate significantly different values (*P*<0.05) by Tukey’s test.

To elucidate whether the increased ATP content in S111-9 via enhanced CEF was used to sequester Na^+^ in the vacuole, V-ATPase activity was determined in the absence and the presence of 100 μM TTFA. As shown in Fig. 8A, the V-ATPase activity was increased significantly under salt stress in both varieties (Fig. 8A). Meanwhile, the expression of V-ATPase subunit A and B genes was increased significantly after salt stress (Supplementary Fig. S4 at *JXB* online). Additionally, in the presence of 100 μM TTFA, the V-ATPase activity was decreased compared with that in the absence of 100 μM TTFA (Fig. 8B). All of these findings supported that the increased ATP content via enhanced CEF might be associated with the sequestering of Na^+^ in the vacuole.

### Changes in expression of genes related to Na^+^, K^+^, and Ca^2+^ transport in mesophyll cells

**Fig. 9.**
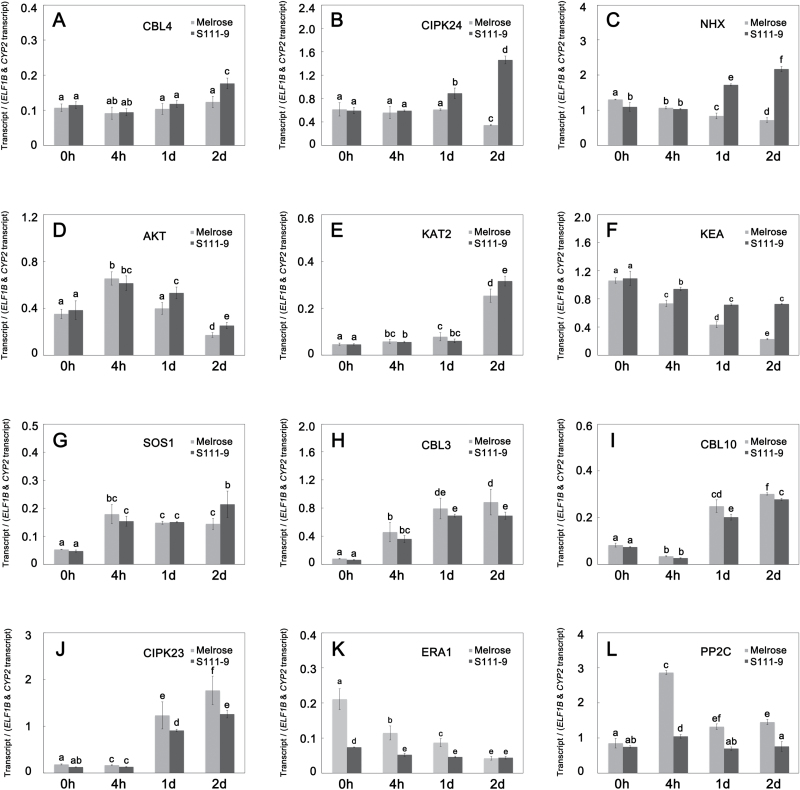
Salt stress-induced expression of genes related to Na^+^ transport. Transcript levels of salt stress-related genes, relative to *ELF1B* and *CYP2* expression, in the leaves of plants treated with 150mM NaCl for 0h, 4h, 1 d, and 2 d (mean ±SD, *n*=3). Different letters indicate significantly different values (*P*<0.05) by Tukey’s test; *n* refers to the number of biological replicates. *NHX*, *KEA*, *SOS1*, *ERA1*, and *PP2C* are Na^+^ transport-associated genes. *NHX* and *SOS1* are involved in the accumulation of Na^+^ in vacuoles and in shoots, respectively. *KEA* is involved in regulating chloroplast function. *ERA1* and *PP2C* function in the regulation of photosynthesis. *AKT* and *KAT2* are K^+^ transport-associated genes, and both of them are related to K^+^ influx. *CBL4*, *CBL3*, and *CBL10* are calcineurin B-like protein genes (CBLs); *CIPK24* and *CIPK23* are CBL-interacting protein kinase genes; all of them are Ca^2+^ transport-associated genes that may assist in Na^+^ transport regulation by Ca^2+^ concentration in the cell.

To assess which Na^+^, K^+^, and Ca^2+^ transport-related genes would respond to salt stress, transcript levels were measured by reverse transcription and quantitative PCR (RT–qPCR) for a series of Na^+^, K^+^, and Ca^2+^ transport-related genes. Among these genes, *CBL4* (calcineurin B-like protein 4), *CIPK24* (CBL-interacting protein kinase 24), and *NHX* (Na^+^/H^+^ antiporter) were significantly up-regulated in response to salt stress in S111-9, but they were unchanged and even down-regulated in Melrose (Fig. 9A–C). Transcript levels of *AKT* (Arabidopsis K^+^ transporter), *KAT2* (K^+^ channel in Arabidopsis thaliana 2), *KEA* (K efflux antiporter), and *SOS1* (salt overly sensitive 1) in S111-9 were also significantly higher than those in Melrose after 2 d salt stress (Fig. 9D–G). Therefore, these genes might be partly positively regulated by salt stress. Transcript levels of *CIPK24* and *NHX* were ~4- and 3-fold higher, respectively, in S111-9 than in Melrose (Fig. 9B, C), which further indicated that *CIPK24*, which is involved in Ca^2+^ transport, and *NHX*, which is involved in Na^+^ transport and accumulation in vacuoles, could directly respond to salt stress in S111-9. In addition, transcript levels of *CBL3* (calcineurin B-like protein 3), *CBL10* (calcineurin B-like protein 10), and *CIPK23* (CBL-interacting protein kinase 23) increased significantly in both varieties after salt stress, but their transcript levels were significantly higher in Melrose than in S111-9 (Fig. 9H–J). Collectively, these results revealed a greater difference between two varieties in transcription of key genes related to Na^+^, K^+^, and Ca^2+^ transport, which could be a response to salt stress.

## Discussion

### Salt stress induces an increase in NDH complex levels and CEF activity

The role of the NDH complex in adaptation to salt stress was reported in studies of an *ndhB*-inactivated mutant of the cyanobacterium *Synechocystis* sp. PCC 6803 (Tanaka *et al.*, 1997), which showed that enhanced CEF from the cytosol to PSI via NDH is essential for the adaptation of cyanobacteria to salt stress. A previous study demonstrated that salt stress could induce significant up-regulation of CEF in soybean (Yang *et al.*, 2007). In addition, the *hcef1 crr2-2* double mutant, which is deficient in thylakoid NDH complex and FBPase, is highly sensitive to light and lacks elevated CEF1, further suggesting that NDH might play a direct role in catalysing or regulating CEF (Livingston *et al.*, 2010*a*, *b*). The reduction of P700^+^ has been taken as a good indicator of NDH activity (Shikanai *et al.*, 1998; Horvath *et al.*, 2000; Rumeau *et al.*, 2007), and the present results suggested that salt stress induces a remarkable increase in NDH activity in S111-9, greater than that in Melrose (Fig. 3).

The results show that salt stress induced increases in NDH-H at the mRNA and protein levels under from 4h to 1 d salt stress in both varieties (Fig. 5). NDH-B protein levels in Melrose markedly decreased on the second day after salt treatment, while NDH-B protein levels remained high in S111-9. The change of NDH-H expression corresponds to the rise in the transient increase in Chl fluorescence after turning off actinic light and the dark re-reduction of P700^+^ in both varieties, implying that NDH-H may play an important role in regulation of CEF under salt stress. Many studies have demonstrated that NDH-B is essential for stabilizing other subunits (Hashimoto *et al.*, 2003; Munshi *et al.*, 2005) and that NDH-H is unstable without other subunits (Hashimoto *et al.*, 2003; Kotera *et al.*, 2005; Rumeau *et al.*, 2007). Additionally, Takabayashi *et al.* (2009) reported that in *ndf* (NDH-dependent CEF) mutants, the amount of NDH-H subunit was greatly decreased and the loss of NDH activity was caused by a defect in accumulation of NDH complex. This further supports the idea that accumulation of both NDH-B and NDH-H can enhance the accumulation and activity of the NDH complex under salt stress. Together, all of these findings suggest that salt stress induces an increase in the amount of the NDH complex, which contributes to enhanced NDH-dependent CEF activity.

### Enhanced NDH-dependent CEF promotes increased ATP contents

Leaf ATP content in light is balanced between ATP production and ATP consumption. Salt stress results in a much greater decrease of the net photosynthetic rate in salt-sensitive Melrose than in salt-tolerant S111-9 plants (Yang *et al.*, 2007; He *et al.*, 2014), indicating that ATP consumption for carbon assimilation should be lower in Melrose than in S111-9 upon light illumination. However, ATP accumulation in S111-9 was much higher than in Melrose (Fig. 4B), suggesting that there is a supplementary pathway producing more ATP in S111-9 than in Melrose under light conditions. The present results showed a greater increase in Chl fluorescence after turning off actinic light (Fig 3A, B) and faster P700^+^ re-reduction (Fig. 3C), reflecting enhanced CEF activity, in S111-9 compared with Melrose under salt stress. Phosphorylation of light-harvesting complex II induced by NaCl and light in salt-tolerant soybean (Lu *et al.*, 2008) supports the idea that the increased absorbed light energy is distributed to PSI and enhances CEF activity. In addition, in the presence of TTFA, the reduction in *t*_1/2_ of P700^+^ re-reduction suggested that CEF activity was decreased, which further resulted in the decrease in ATP accumulation (Figs 3D, 4D). Therefore, the increased CEF activity in light is likely to be the main source of the increased accumulation of ATP in S111-9 compared with Melrose. Although the increase in Chl fluorescence and faster P700^+^ re-reduction also occurred in Melrose upon illumination under salt stress, ATP accumulation decreased as salt stress continued (Figs 3, 4). This may be partly explained by damage to the chloroplasts (He *et al.*, 2014). Moreover, some studies have reported that expression of mitochondrial uncoupling proteins, which can uncouple the electrochemical gradient from ATP biosynthesis and dissipate energy as heat, can be induced by abiotic stress and help plants adapt to abiotic stress conditions (Laloi *et al.*, 1997; Barreto *et al.*, 2014). Both leakage of photosynthetic membranes and activity of uncoupling proteins could reduce the production of ATP, while photosynthetic electron transport occurred normally, or even faster than usual. The co-localization of Na^+^ staining and chloroplasts showed that more chloroplasts contained Na^+^, suggesting more severe damage of photosynthetic membranes, in Melrose than in S111-9 (Fig. 7D, H), which could uncouple election transport from photophosphorylation.

In plants, the majority of photosynthetic electron transport follows the linear (or non-cyclic) pathway passing from water through PSII, the cytochrome *b*_*6*_ *f* complex, and PSI to Fd, and thereafter, for most electrons, to NADP. Linear election flow results in the formation of ATP and reductant, while cyclic electron transport results only in the formation of ATP with no net formation of reductant (Foyer *et al.*, 2012). Although PGR5-dependent CEF and NDH-dependent CEF are partially redundant pathways, the PGR5 pathway might contribute to maintaining the steady state of overall photosynthesis, and the NDH pathway might play a vital role under environmental stresses (Munekage *et al.*, 2004; Johnson *et al.*, 2014). Moreover, the CEF pathway increases the transmembrane proton gradient, which in turn allows for increased biosynthesis of ATP (Kramer *et al.*, 2004), and enhanced CEF contributes in particular to the generation of extra ATP in cyanobacteria (Mi *et al.*, 1995) and higher plants (Asada *et al.*, 1993). Furthermore, C_4_ plants display higher CEF activity than C_3_ plants, and this activity is suggested to be important for the production of ATP required for C_4_ metabolism (Nakamura *et al.*, 2013). These findings also imply that the enhanced CEF activity actually could promote the increase in ATP content. Additionally, the dark respiration rate indicated that accumulation of ATP in S111-9 was not attributed to respiration (Supplementary Fig. S5 at *JXB* online). Furthermore, some studies have reported that an indirect transport of ATP occurred via the DHAP/3-PGA shuttle, which implies that ATP could be exported from the chloroplast to the cytosol. (Heldt *et al.*, 1991; Weber and Linka, 2011). Therefore, it also indicated that accumulated ATP in chloroplast could be utilized by V-ATPase in cytosol.

It was found that the accumulation of the NDH complex (estimated by NDH-H and NDH-B levels) in S111-9 was greater than that in Melrose, as NDH-H and NDH-B increased ~2-fold compared with Melrose (Fig. 5A,C) after 2 d salt stress. In addition, CEF activity in S111-9 was also significantly higher compared with Melrose after salt stress (Fig. 3). Thus, in S111-9, the increase in CEF activity is probably due to increased accumulation of the NDH complex relative to that in Melrose. Also, CEF activity was higher in *Flaveria brownii* (a C_4_-like plant) than in *Flaveria ramosissima* (a C_3_–C_4_ intermediate plant), which is probably due to the increased amount of NDH complex (Nakamura *et al.*, 2013). This further supports the idea that salt stress induces the enhancement of NDH-dependent CEF activity, which could contribute to increasing the ATP content in illuminated leaves.

### Increased ATP content and expression of genes relevant to Na^+^ influx or sequestering Na^+^ in vacuoles relate to the distribution of Na^+^ and salt tolerance

When plants are exposed to stress conditions such as high temperature, drought, and salt, ATP is required in a variety of processes necessary for adaptation of plant cells to stress conditions (Burrows *et al.*, 1998; Yang *et al.*, 2007). For example, the compartmentation in vacuoles and export from organelles and cells of toxic ions, the re-establishment of cellular homeostasis, and the uptake and/or biosynthesis of compatible solutes all utilize ATP (Hasegawa *et al.*, 2000; Qiu and Lu, 2003). Moreover, it is generally accepted that CEF makes an important contribution to the overall regulation of electron transport in response to environmental variation and metabolic cues, even though the nature of these pathways and their precise functions remain controversial (Foyer *et al.*, 2012). It is therefore believed that switching between cyclic and non-cyclic pathways provides a degree of flexibility in the production of ATP and that plants can adjust the relative rate of ATP production to meet the demands of metabolism. Mining published data on transcriptome profiles of C_3_ and C_4_ leaves from plants grown under high atmospheric CO_2_ compared with those grown with ambient CO_2_ revealed that transition to higher photorespiratory conditions in C_3_ plants enhances the expression of genes associated with NDH-dependent CEF pathways (Prins *et al.*, 2011; Queval and Neukermans, 2012), consistent with a higher ATP requirement. Moreover, several mutants with reduced V-ATPase activity appeared to be hypersensitive to Na^+^, supporting the notion that increased V-ATPase activity is required for Na^+^ compartmentalization under salt stress (Batelli *et al.*, 2007; Tang *et al.*, 2012). The present results demonstrated that the increased ATP content via CEF was positively related to V-ATPase activity (Figs 4, 8), further supporting the idea that accumulated ATP was used to sequester Na^+^ in vacuoles by the hydrolysis of V-ATPase.

**Fig. 10.**
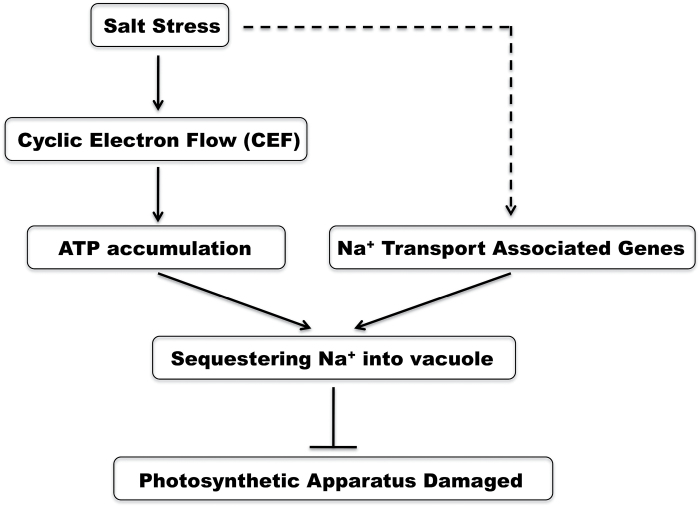
Model for salt tolerance in soybean. Salt stress induces the enhancement of cyclic electron flow (CEF) and up-regulated expression of Na^+^ transport-associated genes. The enhanced CEF promotes increases in ATP content in the light. The increased ATP content in light and the up-regulation of Na^+^ transport-associated genes facilitate sequestering of Na^+^ into vacuoles, which ultimately protects the photosynthetic apparatus from damage. Solid arrows indicate direct regulatory relationships, and the dotted arrow indicates a regulatory pathway.

Blocking Na^+^ influx and sequestering Na^+^ in vacuoles function as important mechanisms for salt tolerance. Some studies have shown that the CBL10–CIPK24–NHX complex and the CBL4–CIPK24–SOS1 complex play vital roles in Na^+^ transport in root cells (Zhu, 2001; Kim *et al.*, 2007). The transcript levels of genes encoding the CBL10–CIPK24–NHX complex at the vacuolar membrane and the CBL4–CIPK24–SOS1 complex at the plasma membrane were remarkably up-regulated in leaves of S111-9 after 2 d salt stress, compared with those of Melrose (Fig. 9A–C, G, I). Thus, in S111-9, the increases in the transcript abundance of genes encoding the CBL10–CIPK24–NHX complex and the CBL4–CIPK24–SOS1 complex points to an important role for these complexes in adjusting Na^+^ transport and compartmentation in mesophyll cells of soybean. The results also suggest that the CBL4–CIPK24–SOS1 complex might contribute to the retrieval of Na^+^ in S111-9 (Figs 2, 9). Furthermore, the transcript levels of *ERA1* (enhanced response to abscisic acid) and *PP2C* (protein phosphatase 2C), which positively and negatively influence photosynthesis, respectively, were examined, and are basically consistent with the decrease in photosynthetic rate under salt stress (He *et al.*, 2014). *KEA* affects chloroplast function and integrity, and is involved in regulating the plastid K^+^ content, the chloroplast osmotic potential, and stromal pH (Kunz *et al.*, 2014). The transcript level of *KEA* was consistent with the likely degree of injury of chloroplasts by Na^+^ entry in both varieties (Figs 7, 9F). Overall, the enhanced abundance of transcripts encoding components of the NDH complex suggested that photosynthetic control of gene expression enhances CEF to meet the increased ATP demand of soybeans under salt stress conditions (Fig. 10). Moreover, transcripts associated with Na^+^, K^+^, and Ca^2+^ transport responded to salt stress, consistent with the expected alterations in supply and demand of ATP related to NDH-dependent CEF activity.

In conclusion, it was demonstrated that in salt-tolerant soybean, salt stress accelerates CEF by a mechanism involved in NDH expression and increased accumulation of ATP in light. The expression of genes associated with Na^+^ transport was also up-regulated. Based on the present results, a model is proposed in which more Na^+^ is sequestered into the vacuoles of mesophyll cells by those gene products, utilizing the energy from ATP, thus alleviating the damage to the photosynthetic apparatus under salt stress conditions (Fig. 10).

## Supplementary data

Supplementary data are available at *JXB* online.

Figure S1. Effects of salt stress on relative leaf weight.

Figure S2. Effects of salt stress on K^+^/Na^+^ ratio in leaf, stem, and root.

Figure S3. Relative fluorescence intensity in vacuoles.

Figure S4. Salt stress-induced expression of vacuolar H^+^-ATPase subunit genes.

Figure S5. Dark respiration rate in intact leaf.

Table S1. Linear correlation analysis of Na^+^, K^+^, and Ca^2+^ concentrations in Melrose and S111-9

Table S2. Primers for RT-qPCR.

Supplementary Data
