# Supplementary material for: Increasing cyclic electron flow is related to Na+ sequestration into vacuoles for salt tolerance in soybean
Source: J Exp Bot. 2015 Aug 14;66(21):6877–89. doi: 10.1093/jxb/erv392 (PMC4623694; doi:10.1093/jxb/erv392)
Supplement: Supplementary Data [file supp_66_21_6877__index.html]

Increasing cyclic electron flow is related to Na+ sequestration into vacuoles for salt tolerance in soybean — Increasing cyclic electron flow is related to Na+ sequestration into vacuoles for salt tolerance in soybean — Supplementary Data 

# Increasing cyclic electron flow is related to Na+ sequestration into vacuoles for salt tolerance in soybean

## Supplementary Data

Data files

- Supplementary Data - Supplementary Data
