# Supplementary material for: Increasing cyclic electron flow is related to Na+ sequestration into vacuoles for salt tolerance in soybean
Source: J Exp Bot. 2015 Aug 14;66(21):6877–89. doi: 10.1093/jxb/erv392 (PMC4623694; doi:10.1093/jxb/erv392)
Supplement: Supplementary Data [file supp_erv392_jexbot153429_file001.pdf]

Supplementary data - figures & tables

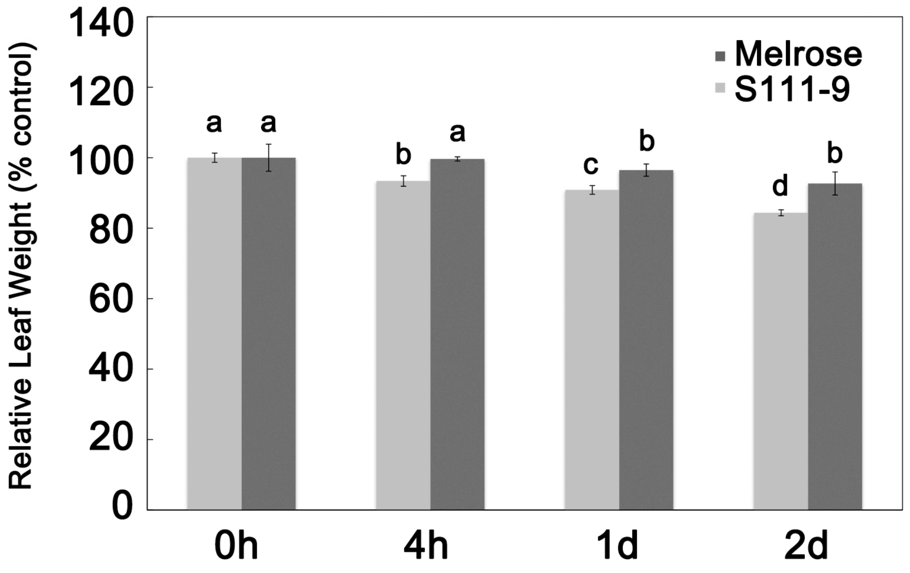

Figure S1. **Effects of salt stress on relative leaf weight.**

The third fully-expanded leaves of Melrose and S111-9 were used in this experiment. The weight of each leaf was measured by analytical balance and the leaf areas were determined by laser portable leaf area meter (*CI-203*) in both varieties at each salt stress time point. Then, the relative leaf weight was calculated based on ratio of leaf weight to leaf area. Each value in this figure is expressed as the percentage of the control. Bars represent the mean ± SD of four independent experiments. Different letters indicate a significant difference among varieties or salt treatment time ($P < 0.05$) by Tukey's test.

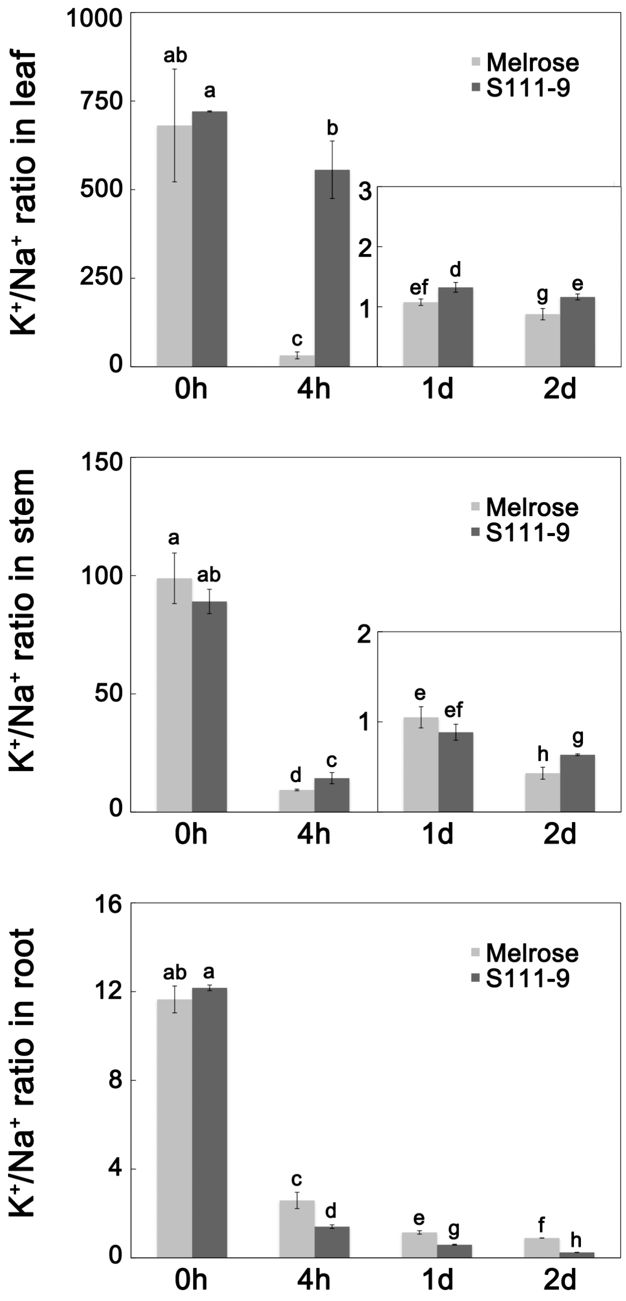

Figure S2**. Effects of salt stress on K^+/Na^+ ratio in leaf, stem and root.**

The concentrations of Na^+ and K^+ were measured in dry samples by ICP-MS (DW, dry weight). All data were normalized to calculated weights, as determined with the solution concentrations. The K^+/Na^+ values were calculated by the ratio of the concentration of K^+ to the concentration of Na^+. Bars represent the mean ± SD of at least three independent experiments. Different letters indicate significantly different values ($P < 0.05$) by Tukey's test.

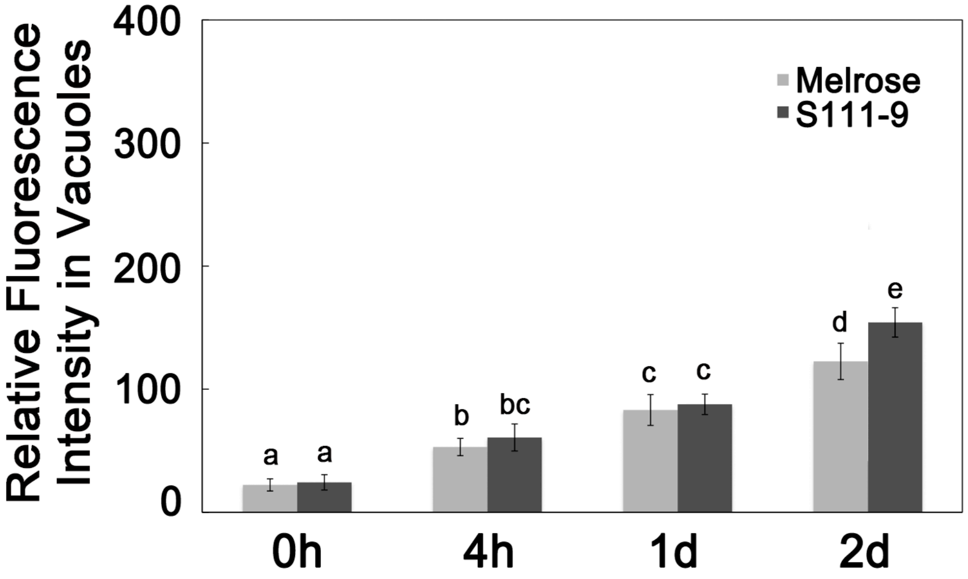

Figure S3. **Relative fluorescence intensity in vacuoles**.

Relative Na^+ signal intensity in vacuoles under 150 mM salt stress. The 63× magnification images stained with CoroNa Green were used to determinate the relative Na^+ concentration. It was analyzed 20 individual cells of palisade layers from both varieties at each salt stress time point. The relative fluorescence intensity of twenty vacuoles was analyzed using Zen2011 software for each salt stress time point in both varieties. The extracellular fluorescence intensity was taken to calculate the background value. And relative fluorescence in vacuoles subtracting the background value finally determined the relative Na^+ concentration in vacuoles. Bars represent the mean ± SD of four independent experiments. Different letters indicate significantly different values ($P < 0.05$) by Tukey's test.

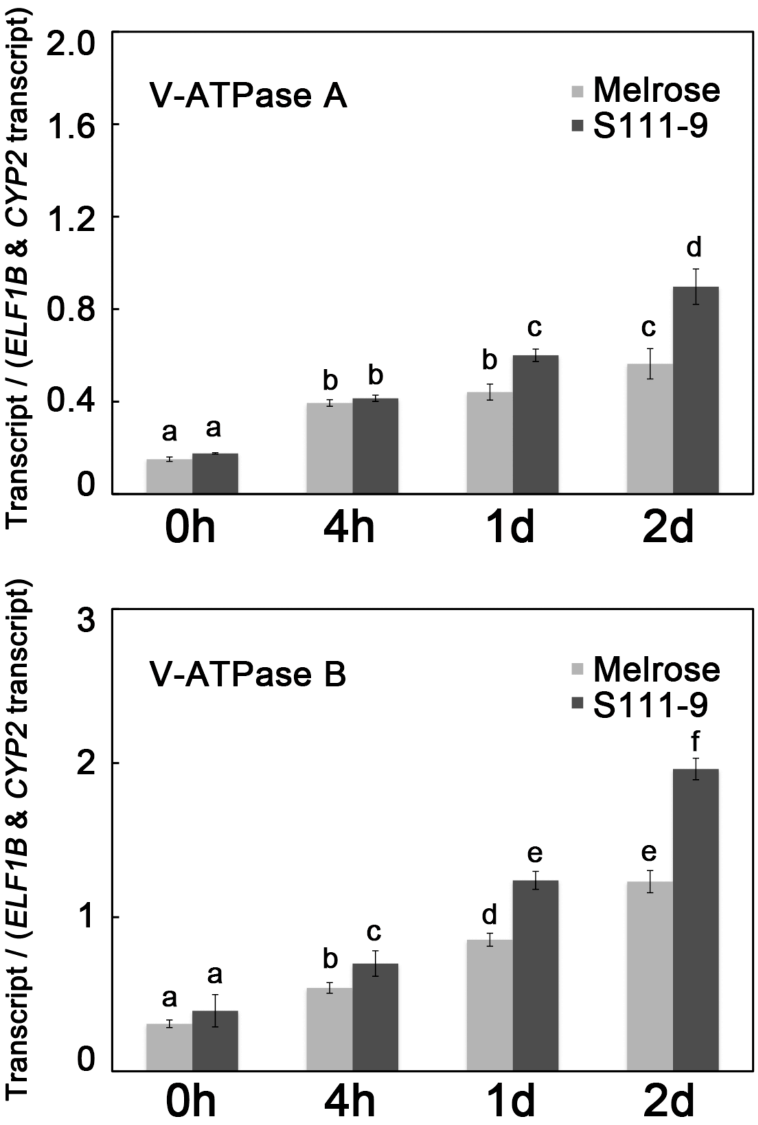

Figure S4. **Salt stress-induced expression of vacuolar H^+-ATPase subunit genes.** Transcript levels of salt stress-related genes, relative to *ELF1B* and *CYP2* expression, in the leaves of plants treated with 150 mM NaCl for 0 h, 4 h, 1 d and 2 d (mean ± SD, n = 3). Different letters indicate significantly different values ($P < 0.05$) by Tukey's test, n refers to number of biological replicates. V-ATPase A and V-ATPase B represent vacuolar H^+-ATPase subunit A and B gene, respectively.

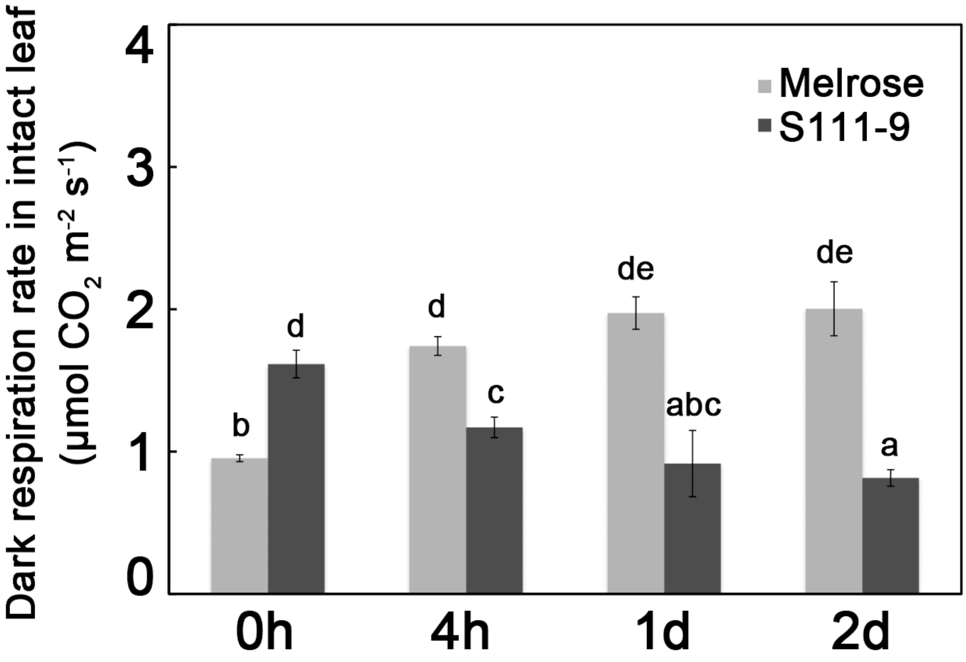

Figure S5. **Dark respiration rate in intact leaf.**

Dark respiration rate in intact leaf was measured as CO_2 efflux from equivalent leaves to those used for intact leaf using a *Licor*-6400 (LICOR Inc. Lincoln NE, USA) equipped with a LED red blue light source. All measurements were carried out at photon flux density (PFD) of 0 mmol m^{-2} s^{-1}, a leaf temperature of 25 °C and CO_2 of 400±5 mmol mol^{-1} in the sample chamber and leaves were adapted in dark for 5 min prior to measurement (mean ± SD, n = 3). Different letters indicate significantly different values ($P < 0.05$) by Tukey's test, n refers to number of biological replicates.

Table S1. Linear correlation analysis of Na^+, K^+ and Ca^{2+} concentrations in Melrose and S111-9

Bold values indicate significant correlations (***R^2****>0.50*) among variables from Na^+, K^+ and Ca^{2+} concentrations based on Pearson's correlation at $P <$ 0.05 (n = 12). Detailed data from Figure 2 was used for correlation analysis.

		Na/K		Na/Ca		K/Ca	
		Equation	R^2 values	Equation	R^2 values	Equation	R^2 values
Leaf	Melrose	y=-0.224x+769.83	**0.7221**	y=-0.0052x+339.85	0.0013	y=-0.1579x+448.06	0.0854
	S111-9	y=-0.0084x+657.89	0.0015	y=0.0769x+306.34	0.5387	y=-0256x+494.33	0.2834
Stem	Melrose	y=-0.4584x+903.67	**0.8452**	y=-0.0762x+327.46	**0.5607**	y=0.1717x+173.36	**0.7073**
	S111-9	y=-0.1055x+787.29	0.2132	y=-0.0929x+338.19	**0.7999**	y=0.2848x+83.99	0.3923
Root	Melrose	y=0.0849x+745.99	0.0237	y=-0.0296x+111.99	**0.6855**	y=-0.038x+124.98	0.2874
	S111-9	y=-0.5234x+625.68	**0.8255**	y=-0.0236x+130.22	0.3841	y=0.0216x+111.42	0.1065

Table S2. Primers for qRT-PCR

Gene Name		Sequences (5'-3')
AKT	Forward	CATCTACACTGAACTGGCAACA
	Reverse	TCGCCCAATAAGGAAACG
CBL3	Forward	TTCATCCCAATGCACCAAT
	Reverse	TGCCAGATTCGGCTAGAGTG
CBL4	Forward	ATGGGGTCATTGAGTTCGG
	Reverse	CGTTCAATAAACCCTGTCTGC
CBL10	Forward	CCGAGGATTCTCCATTTAGTG
	Reverse	TGGTGGTCTTCAACAGTGCC
CIPK23	Forward	TAAGCCTCCCGTATTTGAACA
	Reverse	TCCCTCTTCACGCCTCTCTA
CIPK24	Forward	TCGGCAAGTATGAGGTGGG
	Reverse	TCTGTTCAACCATTCTGTGCTG
ERA1	Forward	GAGTCGCAGGTGTTTCAGATTT
	Reverse	GATTAGCGTCCAAGACAGAAAAC
KAT2	Forward	TTATGGCGGTTGAGACGAG
	Reverse	AGATAGTTAAAGCATCCCGCAC
KEA	Forward	TATCTTGATTGGGCCTTATGGT
	Reverse	GAACTAAGCCTTTCAACAGAGAGC
NHX	Forward	GTCTTGTATTTGGGGAGGGT
	Reverse	ATTGTGCTTGCGATAAATAGATAC
PP2C	Forward	AGTTTCATTTCTTCGCTGTCTT
	Reverse	TCATCGTTGATTCCCATTCC
TPK4	Forward	CACCATAGGTTACGGAGACATAG
	Reverse	ATCCAGAACGAAGTTAACAAGC
ELF1B	Forward	GTTGAAAAGCCAGGGGACA
	Reverse	TCTTACCCCTTGAGCGTGG
CYP2	Forward	CGGGACCAGTGTGCTTCTTCA
	Reverse	CCCCTCCACTACAAAGGCTCG
V-ATPase A	Forward	TATGAACTCGTGCGTGTTGGTC
	Reverse	AAAGGCTTGTGCGTCCGTAA
V-ATPase B	Forward	CTGATAATCTTCTTGAGGGTGG
	Reverse	CAATACGAGGAGTGATAATACGC
